# What factors can support students' deep learning in the online environment: The mediating role of learning self-efficacy and positive academic emotions?

**DOI:** 10.3389/fpsyg.2022.1031615

**Published:** 2022-12-12

**Authors:** Jingxian Zhao, Enyun Liu

**Affiliations:** ^1^Shandong Women's University, Jinan, Shandong, China; ^2^SEGi University, Kota Damansara, Malaysia

**Keywords:** deep learning, learning self-efficacy, positive academic emotions, perceived TPACK support, perceived peer support, perceived technical usefulness and ease of use

## Abstract

**Introduction:**

In 2020, COVID-19 forced higher education institutions in many countries to turn to online distance learning. The trend of using online education has accelerated across the world. However, this change in the teaching mode has led to the decline of students' online learning quality and resulted in students being unable to do deep learning. Therefore, the current research, aimed at promoting deep learning in the online environment, constructed a theoretical model with learning self-efficacy and positive academic emotions as mediators, deep learning as the dependent variable, perceived TPACK support, peer support, technical usefulness, and ease of use as independent variables.

**Methods:**

The theoretical model was verified by SPSS26.0 and smartPLS3.0, and to assess the measurement and structural models, the PLS approach to structural equation modeling (SEM) was performed.

**Results:**

The study found that (a) positive academic emotions play a mediating role between perceived TPACK support and deep learning, perceived peer support and deep learning, and perceived technology usefulness and ease of use and deep learning; (b) learning self-efficacy plays a mediating role between perceived TPACK support and deep learning, perceived peer support and deep learning, and perceived technology usefulness and ease of use and deep learning.

**Discussion:**

The findings of this study fill the gaps in the research on the theoretical models of deep learning in the online environment and provide a theoretical basis for online teaching, learning quality, and practical improvement strategies.

## Introduction

In 2020, COVID-19 forced higher education institutions in many countries to turn to online distance learning, and the trend of using online education has accelerated worldwide (Aguilera-Hermida et al., 2021) with most universities providing distance learning through an online learning system (Aldhahi et al., 2021). However, this change in teaching mode has led to a decline in online learning quality and the problem that students cannot carry out deep learning (Gaeta et al., 2021; Zhang et al., 2021). Moreover, in the current online learning environment, many students are unable to retain attention for long-term online learning or deep thinking, which leads to insufficient online deep learning, mainly manifested in insufficient deep learning behavior, no deep thinking and processing of knowledge to apply theories and concepts to real problems, and no long-term memory of knowledge (Simamora, 2020; Walters et al., 2022). Therefore, many scholars suggested that more studies should be conducted about improving online deep learning (Martinho et al., 2018; Aderibigbe, 2021). Deep learning includes critical thinking, combining what a student is learning with what he or she already knows, and creating new connections and concepts (Ramsden and Entwistle, 1981; Marton and Säljö, 1997; Biggs, 1999). Dummer et al. (2008) believe that deep learning also includes reflective learning, critical thinking, active learning, and other dimensions involving the fine cognitive processing of stimuli, which is helpful in understanding and making information meaningful to learners and producing more lasting and more substantial memory traces. Deep learning is essential not only as a learning method but also as a learning result (Entwistle, 1990; Marton and Säljö, 1997; Biggs, 1999). Through deep learning, students can establish a connection with the original knowledge, form a new knowledge framework, and form critical and reflective thinking and active learning behaviors. In addition, students can produce high-quality learning results (Hall and Ramsay, 2004; Nelson Laird et al., 2014). Therefore, how to improve students' deep learning level in the online environment is a critical factor in improving students' online learning quality.

Currently, the research is based on the concept put forward by Biggs (1999), which posits that students learn for understanding, mainly representing the critical understanding of the learning content, highlighting the connection between prior knowledge and experience, and paying attention to logical relationships and evidence for conclusions in the online learning environment. According to the control value theory of Pekrun et al. (2007), academic emotion, as an intermediary, will affect the learning results and engagement (Pekrun et al., 2007; Yu et al., 2020). Furthermore, according to the theory of self-efficacy put forward by Bandura (1986, 1997a), when students' self-efficacy is high, it will positively impact students' learning results and engagement (Bandura, 1997b; Klassen, 2014). Therefore, the current research tookt the online education environment as the research background, with positive academic emotions and learning self-efficacy as the mediating variables, and explored the factors influencing students' positive academic emotions and learning self-efficacy to improve students' deep learning in an online environment.

**Figure 1:**
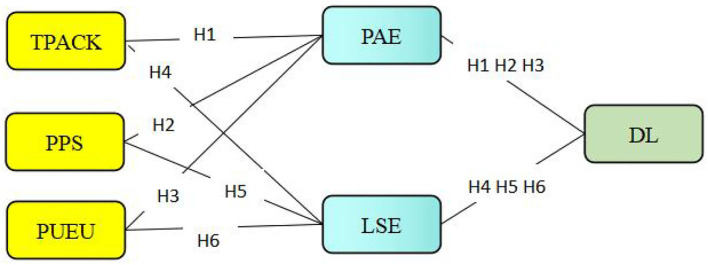
The proposed model.

In previous research, the factors that influence learning self-efficacy or academic emotion in the online environment include teachers' classroom support (Trigueros et al., 2020), family environment (Gaeta et al., 2021), online environment (Parker et al., 2021), learners' attitude toward online teaching or familiarity with online learning equipment (Cussó-Calabuig et al., 2018). However, there is no research on the influence of these three variables, teacher's technological pedagogical content knowledge (TPACK) support, peer support, and perceived usefulness and ease of use of online technology, on students' learning self-efficacy and positive academic emotions in the online environment. Therefore, the current research puts forward the following model assumptions based on the control value theory (Pekrun et al., 2007) and the self-efficacy theory (Bandura, 1997a) (shown in Figure 1):

Hypothesis 1. Positive academic emotions mediate between perceived teachers' TPACK support and deep learning;
Hypothesis 2: Positive academic emotions mediate between students' perceived peer support and deep learning;
Hypothesis 3: Positive academic emotions mediate between students' perceived usefulness and ease of use of technology and deep learning;
Hypothesis 4: Learning self-efficacy plays a mediating role between perceived teachers' TPACK support and deep learning;
Hypothesis 5: Learning self-efficacy mediates students' perceived peer support and deep learning;
Hypothesis 6: Learning self-efficacy mediates between students' perceived usefulness and ease of use of technology and deep learning.

## Literature review

### Deep learning

The first mention of deep learning by researchers was in the 1970s; Marton and Säaljö (1976) proposed that deep learning should emphasize the understanding and memory of learning content. Subsequently, many scholars constantly expanded the concept of deep learning and put forward that deep learning is the process of organizing, synthesizing, and transferring concepts (Biggs, 1970, 1978, 1979, 1987, 1999; Marton and Säljö, 1997; Prosser, 1998; Mahat et al., 2018; Frey et al., 2017), while shallow learning, as opposed to deep learning, is considered as a learning process without active personal participation (Entwistle et al., 2001; Bevan et al., 2014). Deep learning is also related to analytical skills, cross-reference, imagination reconstruction, and independent thinking (Warburton, 2003), while shallow learning usually emphasizes rote learning (Chin and Brown, 2000; Warburton, 2003; Fredricks and Blumenfeld, 2004). Moreover, surface learning is mainly external-centered, which is the tacit acceptance of information and memory, regarded as an isolated and unrelated fact where the focus of learning is to recall and repeat information, leading to the superficial retention of examination materials (Biggs, 1999). Students who use the superficial learning method only aim to acquire enough knowledge to complete tasks or pass courses (Filius et al., 2019). Through deep learning, students learn with the intention of understanding and constructing meaning to do critical thinking, connect new ideas with previous knowledge, and find relationships among materials, to form long-term memory and deep understanding of knowledge (Biggs, 1999; Booth and Luckett, 1999; Trigwell and Prosser, 1999; Chin and Brown, 2000; Akyol, 2011; Pegrum and Bartle, 2014). Therefore, students' deep learning status can represent students' comprehensive learning quality in the online environment. The current research takes online deep learning as the dependent variable, and its primary purpose is to explore how to improve students' deep learning quality in the online learning environment. This research is based on Biggs's concept of deep learning 1970; 1978; 1979; 1999, which is defined as the process of students' deep understanding of knowledge and concepts and the resulting ability to transfer, use, think deeply, and organize comprehensively.

### The mediating role of positive academic emotions

According to the evidence from brain science, the brain regions related to cognition are closely related to emotional processing (Do, 2004; D'Mello and Craig, 2009). In addition, Meyer (2002) proposed that emotional reactions drive the process of learners' problem-solving in complex tasks, so there is a certain degree of correlation between students' deep learning and academic emotions in the online environment. Pekrun et al. (2002, 2007) put forward the control value theory. Academic emotions are positive or negative emotions generated in the academic process, affecting the learning process or results. Positive emotions include happiness, self-confidence, satisfaction, hope, and joy. In contrast, negative emotions include boredom, depression, anxiety, anger, sadness, confusion, and shame (Huang, 2012), and some educational researchers found that academic emotions can affect the use of learning strategies (Marchand, 2012). Pekrun et al. (2012, 2017) also found that positive academic emotions have a positive impact on motivation, meaningful learning strategies, and academic performance, and academic emotions are also the key factors that determine students' learning and persistence in class (Linnenbrink-Garcia and Patall, 2016). However, in the online environment, students have experienced an increase in pressure, anxiety, and depression. Positive academic emotions contribute to teacher-student interaction and academic engagement, while negative emotions hinder academic engagement (Aslan et al., 2020; Odriozola-González et al., 2020; Saravanan et al., 2020; Son et al., 2020) and have an intolerable influence on the learning results (Yu et al., 2020). According to the control value theory, Pekrun et al. (2007) further proposed two types of academic emotions: activity-related emotions and outcome-related emotions. Students experience activity-related emotions in continuous learning activities, such as the pleasure of discovering new knowledge, frustration caused by complex tasks, and boredom when listening to lectures. At the same time, result-related emotions are emotions (such as joy, shame, and pride) related to the achievement of results, such as success and failure, which are very important for students learning participation and development (Pekrun et al., 2012; Nelson Laird et al., 2014; Lee et al., 2021). This research mainly refers to the students' positive academic emotions in deep learning and the positive academic emotions experienced after completing the learning tasks. Positive learning emotion is essential in improving learning participation, engagement, and motivation, but whether it can be used for deep learning in an online learning environment remains to be studied. Therefore, the current research takes students' positive learning emotions as a mediator variable to explore whether the online environment's related factors can affect their online deep learning quality by influencing their internal positive emotional factors.

The current research mainly analyzed the comprehensive factors of teachers, peers, and the technology involved in the online environment and explored whether the online environment can predict students' positive academic emotions from three perspectives: teachers' online TPACK support, perceived peer support level, and perceived technology usefulness and ease of use.

Technological pedagogical content knowledge (TPACK) was first suggested by Mishra (2006). Based on Shulman (1986), the TPACK model had seven knowledge areas: content knowledge (CK) which is the teacher's knowledge about the subject matter to be learned or taught; pedagogical knowledge (PK), which is the teacher's skills and knowledge about the processes and practices or methods of teaching and learning; technological knowledge (TK), which enables teachers to accomplish a variety of different tasks using technological information to develop different ways of accomplishing a given task; pedagogical content knowledge (PCK), which is the selection of appropriate teaching approaches and methods when teaching specific subjects; technological content knowledge (TCK), which is as a combination of technologies and contents relating to understanding how technologies and contents influence and limit each other at the same time; technological pedagogical knowledge (TPK), which includes understanding of how various applications of technological may change teaching and learning; and technological pedagogical content knowledge (TPCK), which is a combination of all the above six components. Interconnection and interactions of all these components create the TPACK framework in the teaching process (Mishra, 2006). In this research, TPACK represents the comprehensive ability of teachers in the online environment. There is relatively little research on academic emotions in the online environment. Wang (2019) used TPACK technology to study the methods of promoting students' emotional intelligence and designed the TPACK curriculum model to adapt to students' emotional intelligence development. At present, there is a lack of research on the influence of perceived TPACK support on students' positive academic emotions in the online environment. Therefore, hypothesis 1 is that positive academic emotions mediate between perceived teachers' TPACK support and deep learning.

Researchers believe peer supports help complete learning tasks (Ladd, 1990, 1999). This research mainly refers to behavior such as peer feedback or other forms that can support and help students in the online environment, and this form of peer support is helpful to students' learning (Filius et al., 2018; Pires et al., 2020). However, there is still a lack of literature on the influence of perceived peer support on positive academic emotions in the online environment. Therefore, hypothesis 2 is that positive academic emotions mediate between students' perceived peer support and deep learning.

The degree of technical support in the online environment significantly influences the positive academic mood (Loderer and Pekrun, 2020). Perceived usefulness and ease of use come from the Technology Acceptance Model (TAM) (Davis, 1986). Perceived ease of use describes individuals' perceived ease of using technology and the degree to which a person believes that using a particular system would be free from effort (Davis, 1986). If the technology is easy to use, then the barriers are conquered, however, if it's not easy to use and the interface is complicated, individuals will have a negative attitude toward it (Davis, 1986). Perceived usefulness refers to the degree to which individuals perceive that using technology will improve their job performance (Davis, 1986). Perceived usefulness and perceived ease of use are critical factors in predicting the attitude toward using technology, which refers to whether or not someone perceives that technology to be useful for what they want to do (Teo et al., 2009). However, there is still a literature gap in the research on the influence of perceived usefulness and ease of use on students' academic emotions in the online environment and whether academic emotions can mediate between perceived usefulness and ease of use and deep learning. Therefore, hypothesis 3 is put forward: positive academic emotions mediate between students' perceived technology usefulness and ease of use and deep learning.

### The mediating role of learning self-efficacy

Self-efficacy is when “people believe that they have the ability to produce expected results through their own actions” (Bandura, 1997a). It also influences academic motivation, learning, and achievement (Schunk, 2002) and predicted academic achievement (Bandura, 1986, 1997a; Lane and Lane, 2004). Many researchers investigated the influence of self-efficacy on learning in an online learning environment (Hung et al., 2010; Wei, 2020). For instance, learning self-efficacy is positively correlated with academic achievement in an online learning environment (Hodges, 2008; Tsai et al., 2011), which has a significant impact on learning performance and results (Dray et al., 2011; Junco, 2012; Klassen, 2014). However, students' network self-efficacy affects their academic performance (Chang et al., 2014), and self-efficacy can predict successful online learning experiences and satisfaction (Tsai et al., 2020). Some studies also showed that students with low self-efficacy may invest less in challenging learning environments (Schunk, 1991; Chemers and Hu, 2001). These studies illustrate the relationship between self-efficiency and the learning process or learning results; learning self-efficacy and achievement, learning motivation, and learning performance correlates. However, no research exists to explore the relationship between self-efficiency and deep learning. Therefore, the current research takes learning self-efficacy as a mediator variable to explore whether teachers, peers, and technology-related factors in the online learning environment can influence online deep learning through learning self-efficacy to improve the quality of deep online learning of students.

Many scholars studied the factors influencing students' learning self-efficacy in the online environment, such as the differences in self-efficacy among students with different professional backgrounds, gender, and other abilities (Huang, 2012; Honicke and Broadbent, 2016; Heo and Bonk, 2021), or learners' attitudes toward online teaching or familiarity with online learning equipment being closely related to self-efficacy (Lee, 2010; Cussó-Calabuig et al., 2018). However, currently, there is no study on the influence of students' perception of TPACK support, peer support, and technology and ease of use on students' learning self-efficacy in the online environment.

Current research on TPACK and self-efficacy mainly focuses on the relationship between teachers' self-perceived TPACK and self-efficacy (Lachner et al., 2021; Zimmermann and Melle, 2021). Akturk (2019) took students' academic self-efficacy and teachers' TPACK as independent variables and students' academic achievement as dependent variables. They verified that academic self-efficacy and teachers' TPACK impact students' academic achievement. Currently, there is no research to explore the relationship between perceived TPACK support and deep learning and students' learning self-efficacy in an online environment. Therefore, the current research puts forward hypothesis 4: the students' learning self-efficacy mediates between perceived TPACK support and deep learning in an online environment.

The related research on peer support and self-efficacy shows that peer support could be used as a moderating variable to influence self-efficacy (Zhao, 2021). Self-efficacy can be used as a mediating variable between peer support and adolescent health (Chen and Sun, 2017). There is no research on whether learning self-efficacy plays a mediating role between peer support and deep learning in the online environment. Therefore, the current research puts forward hypothesis 5: learning self-efficacy mediates between perceived peer support and deep learning in the online environment.

Among previous literature, Zainab et al. (2017) studied the relationship between computer self-efficacy and perceived usefulness and ease of use of technology. There is no research on the relationship between perceived technical usefulness and ease of use in the online education environment and learning self-efficacy. Therefore, the current research puts forward hypothesis 6: Learning self-efficacy mediates between students' perceived usefulness and ease of use of technology and deep learning.

## Research methods

### Participants and research context

The data were collected from three universities to ensure the richness and normal distribution of the data (Parsons, 2014). The current research selected 50 students as pilot study participants to test the questionnaire's reliability and validity. Subsequently, 300 students were selected through stratified random sampling from different majors and grades from three local universities in China.

### Measurement instrument

#### Instruments

Because currently there is no relevant questionnaire on the online learning environment relating to the variables being explored in this research, the current research questionnaire was based on the following adapted questionnaire. The primary purpose of the adapted questionnaire was to measure students' perceived teacher's TPACK support, perceived peer support, perceived technology usability and usefulness, learning self-efficacy and positive academic emotion, and deep learning in the online environment. Based on previous studies, items were measured with a 5-point Likert scale (1 = strongly disagree, 5 = strongly agree).

The TPACK questionnaire for an online environment was adapted from Jang and Chen (2013); Dobi Barišić and Divjak (2019); Dong et al. (2020), to measure university students' perceptions of instructors' TPACK. The current research environment mainly focuses on teachers' content knowledge (CK), teaching knowledge (PK), technological knowledge (TK), teaching content knowledge (PCK), technological content knowledge (TCK), technological pedagogical knowledge (TPK), and technological pedagogical content knowledge (TPCK) level perceived by students in the online learning environment. After adapting, a scale of seven factors and twenty-nine specific items was formed, as shown in Table 1.

**Table 1:** Perceived technological pedagogical content knowledge scale.

TK	1	I think most of my teachers know how to solve their own technical problems in online teaching classroom
	2	I think most of my teachers can learn technological easily in online teaching classroom
	3	I think most of my teachers keep up with important new technologies in online teaching classroom
	4	I think most of my teachers know about a lot of different online teaching technologies in online teaching classroom
	5	I think most of my teachers have the technical skills if they need to use technologies in online teaching classroom
CK	6	I think most of my teachers have sufficient knowledge about subject knowledge in online teaching classroom
	7	I think most of my teachers can use a subject knowledge way of thinking in online teaching classroom
	8	I think most of my teachers have various ways and strategies of developing my understanding of knowledge in online teaching classroom
	9	I think most of my teachers can use a historical and theoretical way of thinking in online teaching classroom
PK	10	I think most of my teachers know how to assess student performance in online teaching classroom
	11	I think most of my teachers can adapt students' teaching based-upon what students currently understand or do not understand in online teaching classroom
	12	I think most of my teachers can adapt different teaching style to different learners in online teaching classroom
	13	I think most of my teachers can assess student learning in multiple ways in online teaching classroom
	14	I think most of my teachers can use a wide range of teaching approaches in online teaching classroom
	15	I think most of my teachers are familiar with common student understandings and misconceptions in online teaching classroom
	16	I think most of my teachers know how to organize and maintain online teaching classroom management.
PCK	17	I think most of my teachers can select effective teaching approaches to guide student thinking and learning in specific subject knowledge when they teaching online
	18	I think most of my teachers can select effective teaching approaches to guide student thinking and learning in technological literacy when they teaching online
	19	I think most of my teachers can select effective teaching approaches to guide student thinking and learning in specific major when they teaching online
TCK	20	I think most of my teachers know about how to use online technologies for helping students' understanding subject knowledge.
	21	I think most of my teachers know about technologies that they can use for understanding specific major when they online teaching.
	22	I think most of my teachers know about technologies that they can use for students understanding better of their major knowledge
TPK	23	I think most of my teachers can choose technologies that enhance the teaching approaches for a online lesson.
	24	I think most of my teachers can choose technologies that enhance students' learning for a lesson.
	25	I think most of my teachers can thinking critically about how to use technological in their online teaching classroom
TPCK	26	I think most of my teachers can structure activities to help students to construct different representations of the content knowledge using appropriate online technologies tools
	27	I think most of my teachers can create self-directed learning activities of the content knowledge with appropriate online technologies tools
	28	I think most of my teachers can design inquiry activities to guide students to make sense of the content knowledge with appropriate online technologies tools
	29	I think most of my teachers can design lessons that appropriately integrate content, online technologies and pedagogy for student-centered learning.

The Peer Support Scale perceived by students during online learning is adapted from the Peer Support Questionnaire of Chen (2008) and The Classmate Support Scale (Torsheim et al., 2000). The current research environment mainly refers to students' perception of peer support, help, or feedback in the online learning environment. Five items were adapted to this scale, as shown in Table 2.

**Table 2:** Perceived peer support scale.

1	I think positive feedback from classmates online helps me think and learn
2	In the form of group mutual assistance, I can better master knowledge in online learning
3	Compared to my personal learning style, I prefer to study cooperatively with my classmates
4	I think the support of classmates can make me feel relaxed in the online learning environment
5	When I study with my classmates, I can complete the learning tasks faster

Students' perceived usefulness and ease of use in the online environment are based on the ICT Attitude of Dong et al. (2020). The current research environment mainly refers to students' perception of peer support, help, or feedback in the online learning environment. Five items were adapted to this scale, as shown in Table 3.

**Table 3:** Perceived usefulness and ease of use scale.

1	I can solve most technical problems when using online technologies if I invest the necessary effort.
2	When I am confronted with a problem when using online learning technologies, I can usually find several solutions
3	I am willing to spend more time learning online technological because I find it useful
4	Taking online learning courses will not make me feel embarrassed
5	Learning with online teaching technological makes me feel very comfortable

The Self-efficacy Scale of students in the online environment is mainly adapted from the self-efficacy questionnaire of Sherer et al. (1982). The current research environment mainly refers to the degree to which students believe that they can use online learning technology to solve problems and learn deeply, and five items were adapted as the scale, as shown in Table 4.

**Table 4:** Learning self-efficacy scale.

1	Before the start of each online course, I will make a complete study plan and be sure to complete it
2	I think I can solve the technical problems encountered in online learning
3	I think I can use online technological to search for information related to learning
4	I think I can use online technological to solve the problems I encountered in my studies
5	I think I can think and master in-depth knowledge in an online learning environment

The Positive Academic Emotion in an online environment was adapted from the Student Engagement and Disaffection in School (Skinner et al., 2008) questionnaire to measure students' enjoyment, enthusiasm, fun, pride, and interest in academic emotions in the online learning environment, and six items were adapted as the scale, as shown in Table 5.

**Table 5:** Positive academic emotion scale.

1	I find that learning knowledge can give me a deep sense of personal satisfaction
2	I find that I have to do enough work on a topic so that I can form my own conclusions and then satisfy me
3	I think that once you enter the research of any topic, almost any topic will be very interesting
4	I find most new topics are interesting and I often spend more time trying to get more information about them
5	I find that learning academic topics can sometimes be as fun as reading a good novel or watching a movie
6	I spend a lot of free time to learn about interest topics that have been discussed in different courses

The Deep Learning Scale of the students is the Revised Study Process Questionnaire (R-SPQ; Biggs et al., 2001), which is a modified version of the Study Process Questionnaire (SPQ) based on the concept of deep learning of Biggs (1970, 1978, 1979, 1987, 1999). Biggs' concept of deep learning is defined as the process of students' deep understanding of knowledge and concepts and the resulting ability to transfer, use, think deeply, and organize comprehensively and then adapt to the online environment. A total of five items were selected for the scale, as shown in Table 6.

**Table 6:** Deep learning scale.

1	I can remember facts, opinions or methods in the course and reading materials, and can repeat them in almost the same form
2	I can analyze the basic elements of thought, experience or theory, such as in-depth study of a specific situation and considering its components
3	I can synthesize and organize thoughts, information or experience to form new and more complex explanations and relationships
4	I can make judgments on the value of information, arguments or methods, such as checking how other people collect Internet data, and assess the reliability of their conclusions
5	I can apply theories or concepts to real problems or new situations

#### Pilot study

The pilot study used SPSS26.0 to conduct exploratory factor analysis (EFA) to improve the reliability and validity of the questionnaire and delete unnecessary items. The specific standards are given as follows: the Sphericity Bartlett Test (*p* < 0.500), the Kaiser-Meyer-Olkin (>0.800), the Cumulative Variance Explained (≥50%), the Communalities (≥0.300), and the Eigenvalue (≥1.00) (Hair et al., 2010; Pallant, 2011). Cronbach's Alpha >(0.700) (Hair et al., 2010) for items that did not meet the above criteria were deleted. After testing, the pilot study results showed that the questionnaire had good reliability and validity, and all items met the above criteria in this step. Each scale result is shown in Table 7.

**Table 7:** Pilot study results.

**Scale**	**Cronbach's alpha**	**KMO**	**Sphericity Bartlett test**	**Cumulative variance explained**	**The smallest items communalities**	**Eigenvalue**
Perceived TPACK	0.981	0.796	0.000	78%	0.654	≥1.00
Perceived peer support	0.860	0.804	0.000	86%	0.463	≥1.00
Perceived use and ease of use	0.779	0.834	0.000	63.8%	0.538	≥1.00
Learning self-efficacy	0.891	0.875	0.000	71%	0.549	≥1.00
Positive academic emotion	0.894	0.804	0.000	66%	0.531	≥1.00
Deep learning	0.897	0.801	0.000	72%	0.635	≥1.00

### Data collection and data analysis

In total, 300 questionnaires were collected, of which, 160 were boys (53.3%), and 140 were girls (46.7%). It included 138 freshmen (46.0%), 25 sophomores (8.3%), 133 juniors (44.3%), and 4 seniors (1.3%). Data analyses were performed using Partial Least Squares (PLS). To assess the measurement and structural models, the PLS approach to structural equation modeling (SEM) was performed (Ringle et al., 2005). For the hypotheses testing, the standard PLS algorithm was performed to assess the significance level of the estimates based on 5,000 bootstraps, as suggested by Hair et al. (2011).

## Findings

### Assessment of the measurement model

**Figure 2:**
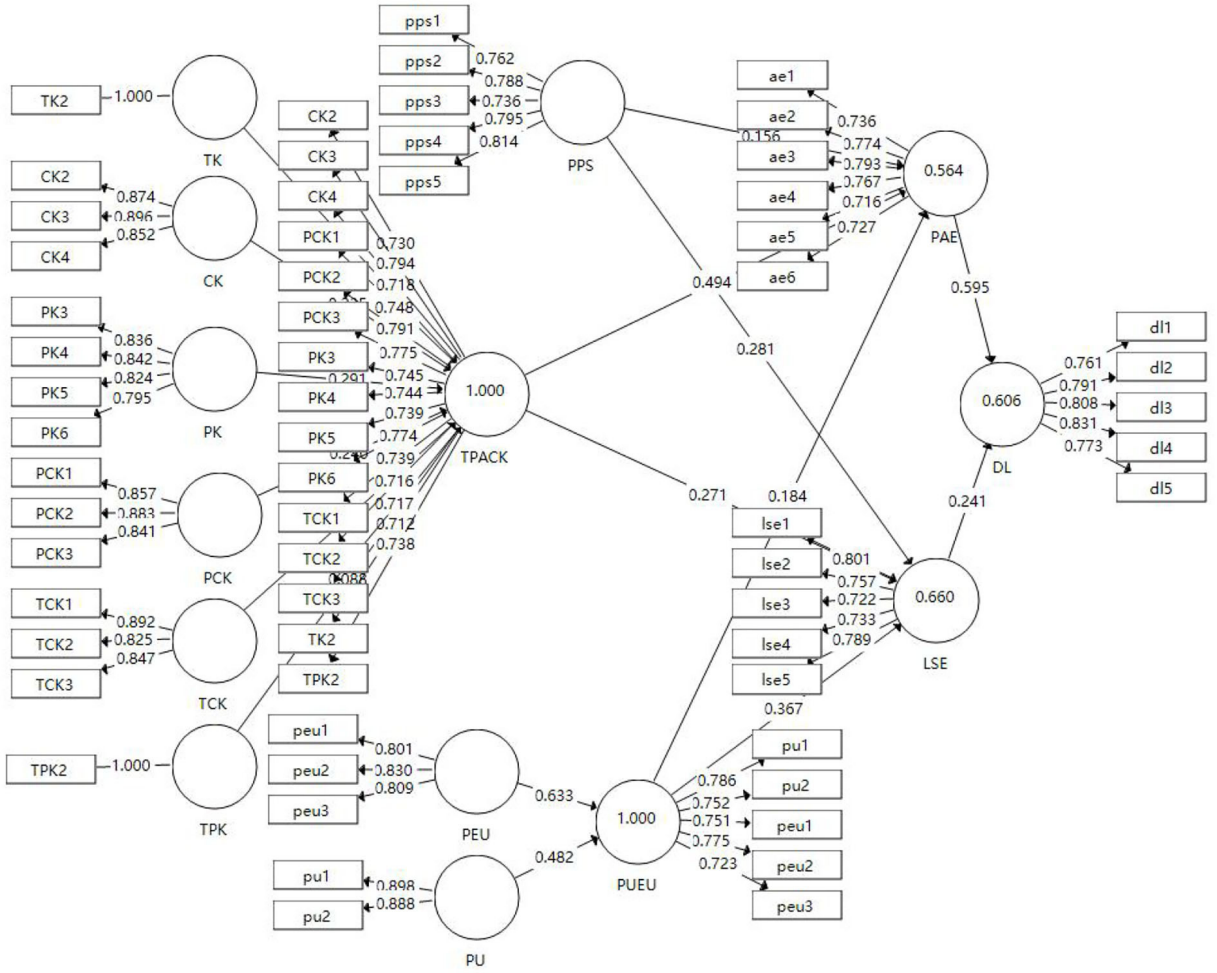
PLS-path analysis of *R*^2^ values (*n* = 300).

A two-step approach was adopted for this study following Anderson and Gerbing (1988) recommendations. The first step was to examine and evaluate the convergent validity and reliability. Convergent validity is achieved when the model satisfies the following criteria. First, loading should exceed 0.7 (Bagozzi and Yi, 1988). Values <0.7 are recommended to be omitted, according to Hair et al. (2014). Second, the composite reliability should exceed 0.7 (Gefen et al., 2000). Finally, Fornell and Larcker (1981) stated that an average variance extracted (AVE) should exceed 0.5. Hence, based on the results, the model met all of the above criteria after deleting some items which loaded <0.7, the remaining items as presented in Table 8, Figure 2.

**Table 8:** Measurement model of PLS.

**Variable**	**Cronbach's alpha**	**rho_A**	**Composite reliability**	**Average variance extracted (AVE)**
DL	0.853	0.854	0.895	0.629
LSE	0.818	0.818	0.873	0.579
PAE	0.847	0.848	0.887	0.566
PPS	0.839	0.844	0.885	0.607
PEU	0.744	0.745	0.854	0.662
PU	0.745	0.746	0.887	0.797
PUEU	0.814	0.816	0.871	0.574
CK	0.845	0.848	0.907	0.764
PK	0.843	0.843	0.895	0.680
TK	1.000	1.000	1.000	1.000
PCK	0.825	0.826	0.895	0.741
TCK	0.816	0.816	0.891	0.731
TPACK	0.943	0.943	0.949	0.556

### Discriminant validity

The following step examines the discriminant validity. Discriminant validity was tested using the HTMT criterion (Henseler et al., 2016), where the discriminant validity is established if the values are <0.90 threshold (Kline, 2011). The research model HTMT value ranged from 0.430 to 0.896. The convergent validity, reliability, and discriminant validity models were qualified based on the evaluations.

### Second-order construct assessment

This study incorporated two evaluations to assess the second-order construct of TPACK and PUEU models (Hair et al., 1998; Wong, 2016; Hair J. F. et al., 2017), which are collinearity issues, as well as the significance of formative indicators. In terms of collinearity, the variance inflation factor (VIF) values for dimensions of TPACK ranged from 1.822 to 3.272, and the variance inflation factor (VIF) values for dimensions of PUEU were 1.558 and below 5, thus indicating satisfactory reliability (Hair et al., 1998; Hair G. T. et al., 2017). The results, therefore, did not indicate a multi-collinearity problem and supported the formative nature. The weight of each dimension was above the recommended value of 0.10 (shown in Figure 2) (Hair et al., 1998; Hair G. T. et al., 2017). All these weights of formative indicators also had significant *t*-values and provided empirical support to retain all the indicators (shown in Figure 2).

### Assessment of the structural model

**Figure 3:**
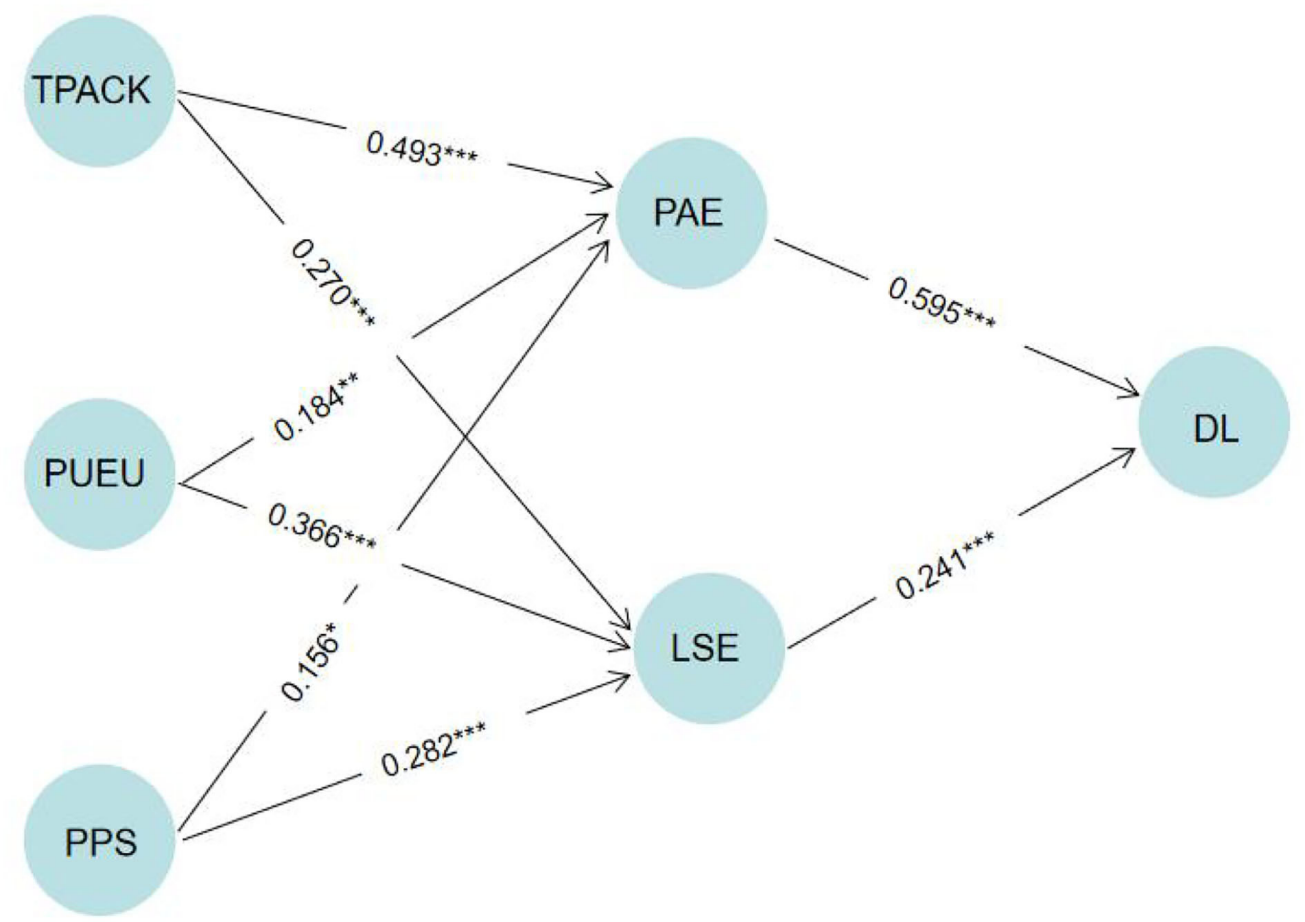
Significance of effects path. *p < 0.05, *t* > 1.645; **p < 0.01, *t* > 2.327; ***p < 0.001, *t* > 3.092.

To test the hypotheses, a bootstrapping procedure with a resampling rate of 5,000 (Hair G. T. et al., 2017) was performed to obtain the Beta value, *t*-values, *p*-values, and bootstrapped confidence intervals. It applied values for a one-tailed *t*-test of 1.645 (significant level = 0.05), 2.327 (significant level = 0.01), and 3.092 (significant level = 0.001) (Hair G. T. et al., 2017). According to the bootstrap process, Table 9 and Figure 3 exposes the existence of influence. Thus, H1 to H6 are supported.

**Table 9:** Significance of effects path (*n* = 300).

**Path**	**Standard path coefficients**	**Sample mean (M)**	**Standard deviation**	***T*-statistics**	***P*-values**	**Results**
TPACK -> PAE -> DL	0.294***	0.295	0.054	5.425	0.000	H1 supported
PPS -> PAE -> DL	0.092*	0.095	0.044	2.113	0.017	H2 supported
PUEU -> PAE -> DL	0.110**	0.107	0.043	2.541	0.006	H3 supported
TPACK -> LSE -> DL	0.065**	0.064	0.025	2.657	0.004	H4 supported
PPS -> LSE -> DL	0.068**	0.070	0.025	2.669	0.004	H5 supported
PUEU -> LSE -> DL	0.088**	0.088	0.028	3.106	0.001	H6 supported

*p < 0.05, *t* > 1.645; **p < 0.01, *t* > 2.327; ***p < 0.001, *t* > 3.092 (one tailed).

### R^2^ and Q^2^ value

The coefficient of determination (R^2^) is the value that measures the prediction accuracy of the model, calculated by the square correlation between the actual value and the predicted value of a specific endogenous structure or dependent variable (Hair et al., 2016). The value range of R^2^ is 0 ~1, and the higher the value is, the higher the prediction accuracy is. An R^2^ value of 0.75 is considered strong, while 0.50 is medium and 0.25 is weak (Hair et al., 2016). The result of R2 was DL = 0.606; LSE = 0.660; PAE = 0.564. Therefore, it can be considered that the data in this study have good prediction accuracy.

Q^2^ can also be used as a criterion for predictive relevance (Stone, 1974). Henseler and Fassott (2009) also pointed out that this measure can be used to evaluate the research model's capability to predict. According to the blindfold procedure, Q^2^ evaluates the predictive validity of a model *via* PLS. Q^2^ values larger than zero indicate exogenous constructs that possess predictive relevance for the endogenous construct, 0.02 as weak; 0.15 as moderate; 0.35 as strong (Hair et al., 2011). In this study, the result of Q^2^ (DL = 0.376; LSE = 0.369; PAE = 0.313) indicated that the research model has excellent predictive relevance.

## Discussion

First, the current research has verified that students' positive academic emotions mediate between perceived TPACK support, peer support, technical usefulness, ease of use, and deep learning in the online environment. This conclusion has also verified the control value theory (Pekrun et al., 2002, 2007) and that academic emotions influence deep learning. Pekrun et al. (2012, 2017) also found the influence of academic emotions on learning motivation and academic achievement. Wang (2019) promoted the development of students' emotional intelligence by using the TPACK technology model. These similar studies also validated the current research conclusions. Moreover, this conclusion filled the gap in the research on the relationship between perceived TPACK support, positive academic emotion, and deep learning in the online environment. It showed that the comprehensive useability of teachers' teaching knowledge, subject knowledge, and online technology in the online environment could affect students' emotions and indirectly affect students' deep learning levels. Therefore, in online teaching, teachers should pay attention to improving their TPACK level, which also plays an essential role in improving students' learning emotion and deep learning. In addition, this study's conclusion fills the gap in the literature between students' perceived peer support and positive academic emotion in the online environment and shows that peer assistance or cooperative learning style are both factors that influence students' positive academic emotions and deep learning. Finally, the research conclusion also fills the gap in the relationship between students' perceived usefulness and ease of use of online technology, positive academic emotions, and deep learning, which also illustrates how improving students' adaptability to technology is one of the effective ways to improve students' deep learning in the current online teaching environment.

Furthermore, the current research has verified that students' learning self-efficacy mediates between perceived TPACK support, peer support, technical usefulness, ease of use, and deep learning in the online environment. This conclusion has verified the theory of self-efficacy (Bandura, 1997a), and students' expectation that self-study will affect their learning results (Bandura, 1986, 1997a). Previous similar research, therefore, validates the current research conclusion. Teachers and peer support can indirectly influence deep learning through self-efficacy (Zhao, 2021). In addition, the current research fills the gaps in the literature on perceived TPACK, peer support, perceived technical usefulness and ease of use, and academic self-efficacy. It explains the comprehensive ability of teachers' professional and technical application and the critical role of peer support and technology platforms for students' deep learning online. Attempts to improve teachers' TPACK level, peer support, and adaptability of online learning platforms are effective measures to improve students' online deep learning in the future.

Finally, in the post-pandemic era, online education has gradually become a routine teaching mode, but the quality of online teaching and learning still needs improvement (Aguilera-Hermida et al., 2021; Aldhahi et al., 2021). This research took positive academic emotions and learning self-efficacy as mediating variables, which showed that paying attention to students' academic emotions and self-efficacy in the online environment is helpful to students' deep learning, thus improving the quality of online learning. This finding is similar to that of previous research emphasizing individual factors (Huang et al., 2019; Huang and Yang, 2020, 2022). This research provides a perspective for improving positive academic emotions and self-efficacy from teachers' TPACK, peer support, and technical usefulness and ease of use. Although the theoretical model has been constructed in this research, and the theoretical research is based on extant literature on the relationships between students' perceived TPACK, peer support, technical usefulness and ease of use, academic emotion, efficacy, and deep learning, as the current research is longitudinal, more related cross-sectional research is needed on how to design specific online teaching modes and improve the applicability of teaching platforms to improve students' online learning quality.

## Limitations

Although this research has verified that perceived TPACK, peer support, technological usefulness, and ease of use can influence positive academic emotion and learning efficacy and then influence deep learning in an online environment from a quantitative perspective, further research is needed drawing from concrete examples of students in online learning which will be more precise. Furthermore, this research mainly focused on investigating deep learning without surface learning as comparable, so further research could investigate surface learning and deep learning in the online environment. Finally, this research ran an EFA test using 50 samples which could have caused some items to not be tested on whether they meet the criteria; thus, further research should collect more samples to address this issue.

## Data availability statement

The raw data supporting the conclusions of this article will be made available by the authors, without undue reservation.

## Ethical statement

Written informed consent to participate in this study was provided by the participants. All procedures performed in studies involving human participants followed the Institutional and National Research Committee's Ethical Standards and the 1964 Declaration of Helsinki and its later amendments or comparable Ethical Standards. The current research institute approved all three Universities' Research procedures, which considered all Ethical issues.

## Author contributions

All authors listed have made a substantial, direct, and intellectual contribution to the work and approved it for publication.
